# Narrative medicine pinpoints loss of autonomy and stigma in Parkinson’s disease

**DOI:** 10.1038/s41531-023-00593-y

**Published:** 2023-11-01

**Authors:** Barend W. Florijn, Raoul Kloppenborg, Ad A. Kaptein, Bastiaan R. Bloem

**Affiliations:** 1 Department of Neurology, Leiden University Medical Center, Leiden, the Netherlands; 2 Department of Neurology, Hague Medical Center Westeinde, The Hague, the Netherlands; 3 Einthoven Laboratory for Vascular and Regenerative Medicine, Leiden University Medical Center, Leiden, the Netherlands; 4 Department of Medical Psychology, Leiden University Medical Center, Leiden, the Netherlands; 5 Radboud University Medical Center; Donders Institute for Brain, Cognition and Behaviour; Department of Neurology, Center of Expertise for Parkinson & Movement Disorders, Nijmegen, The Netherlands

**Keywords:** Parkinson's disease, Neurological manifestations

## Abstract

Parkinson’s disease characteristics can create a self-perceived sense of stigmatization and disapproval by others, thereby affecting self-perceived autonomy. This study investigated the metaphors related to the loss of autonomy and stigma in stories and drawings of Parkinson’s disease. We compare a contemporary first-person illness narrative and -drawing from a person with Parkinson’s disease, with two novels (Jonathan Franzen’s *The Corrections* and Claudia Piñeiro*’s Elena Knows)*, a graphic novel *(*Peter Dunlap-Shohl’s *My Degeneration: A Journey Through Parkinson’s)*, a non-fiction book (Oliver Sacks’ *Awakenings*) and a first-person illness narrative (John Palfreman’s *The Bright Side of Parkinson’s*). Metaphors in the patient narrative, novels, and non-fiction work were reviewed and a list of themes or categorizations common to 2 of the metaphors was generated. Parkinson’s disease metaphors indicate a ‘Parkinson’s prism’ thereby depicting extreme experiences (24.4%) like a ‘fall by mischance’, a ‘tantrum of selfish misery’ or a ‘bottomless darkness and unreality’ (Table 1). Both novels signify a sense of ‘betrayal and disconnection’ in the Parkinson’s disease experience while non-fiction of Parkinsonism depicts a *space* in which one feels ‘caged and deprived’. This makes the Parkinson’s disease narrative a chaos story that could influence the decision to initiate treatment and treatment adherence. We conclude that narrative medicine can help to focus the medical consultations with affected individuals on issues that matter most to them, thereby improving self-perceived autonomy and stigma. As such, it is a critical component of the much-needed move towards personalized medicine in Parkinson’s disease, achieved through the reciprocity of thinking with stories.

## Introduction

Parkinson’s disease is a neurodegenerative disease which prevalence increases with age and it is currently reckoned among the fastest growing neurological diseases in the world (1). The disease is characterized clinically by a combination of motor symptoms (among others bradykinesia, tremor, and rigidity) and nonmotor symptoms (such as changes in mood or sleep) that jointly have a profound impact on a person’s subjective and psychological well-being^1,2^. Particularly the change in physical characteristics, such as the stooped posture, shuffling gait, facial masking or trembling, can create a self-perceived sense of stigmatization and disapproval by others^3^. As such, stigma illustrates how the burden of Parkinson’s disease extends far beyond the well-known physical limitations.

Narrative medicine is an interdisciplinary field that aims to teach both medical students and physicians to practice more competently, by learning the ability to recognize and be motivated by stories of illness^4^. Major theoretical and empirical contributions in narrative medicine have been made by Arthur W. Frank and Arthur Kleinman and *illness narratives* is a core theoretical concept introduced by these two scholars^5,6^. Arthur W. Frank defines three categories of illness narratives which are particularly relevant in the context of this paper. *Restitution stories* attempt to outdistance mortality by rendering illness transitory. *Chaos stories* are sucked into the undertow of illness and the disaster that attend it. *Quest stories* meet suffering head on: they accept illness and seek to use it. The recognition of these stories starts when physicians become adjusted to “thinking with stories” in clinical care^5^ ^(pp. 23-24).^ Thinking with stories is a *narrative competence* that comes instead of thinking 'about stories', thereby allowing the narrative to naturally work on us, instead of conceiving narratives as an object^7^. We previously used novels to provide such a perspective^8–10^ and analyzed the language of illness perceptions in patient stories^11^. It is unknown however, how figurative language and the use of metaphors function in the Parkinson’s disease experience and whether they influence factors such as physician-patient interaction, the decision to initiate treatment, or treatment adherence.

In *illness narratives*, metaphors are frequently part of the language that is used to reveal the particularities of what it is like to be ill^12^. In ‘*Illness as metaphor’*, Susan Sontag however argues that the language of illness is ‘not a metaphor’ and that ‘the healthiest way of being ill is one that is most purified of, and most resistant to, metaphoric thinking’^13^. What particularly enraged Sontag (when she became a cancer patient herself) “was seeing how much the very reputation of this illness added to the suffering of those who have it”. Therefore, metaphors, Sontag argued, tend to “deform the experience” of disease and “inhibit people from seeking treatment early enough, or from making a greater effort to get competent treatment”^14^. In the decade since she wrote Illness as Metaphor, Sontag stated that her aim was ‘to alleviate unnecessary suffering’ because ‘the metaphoric trappings that deform the experience of having cancer have very real consequences: they inhibit people from seeking treatment early enough, or from making a greater effort to get competent treatment.’ Collectively this indicates that metaphors ‘are active in shaping a landscape of practice and inculcating values, rather than being merely descriptive and passive.’^15^.

In contrast, others have argued that metaphors are a form of ‘imaginative reality’^16^ thereby offering ‘a pathway to new understandings’^17^. Martha Holmes stated in her essay ‘After Sontag: Reclaiming Metaphor’ that metaphors ‘decrease suffering rather than add to it’^18^. They “express our own embodiment” thereby “getting us closer to those things we cannot palpate ourselves or see without technology”^18^. As such, “metaphors create relationships” that could “change patients’ and doctors’ attitudes toward embodiment and illness. This could influence “self-diagnosis and the timing of diagnosis, and potentially to change the course of illness and health”^18^. An additional method to examining this resides in patients’ drawings of their illness, the symptoms, and its medical management. Drawings produce data that help in understanding the patient’s answer to the illness, which in turn -if incorporated in the medical management- could improve the quality of the clinical, medical response to the patient’s plight^19^. In the field of Parkinson’s disease however, it is unknown which metaphors are used in the illness narrative to describe the loss of autonomy and the self-perceived sense of stigmatization that may follow the physical attributes of disease nor whether they impact the course of illness.

The objective of this study, is to train both medical students and physicians the ability to recognize the metaphors related to autonomy and stigma in stories and drawings of Parkinson’s disease. Therefore, our aims were threefold. First, we portray the self-perceived sense of stigmatization and loss-of-autonomy-experience in Parkinson’s disease, as represented by a story and -drawing of the illness experience from a person with Parkinson’s disease. Second, we compare this person’s narrative to the metaphors of Parkinson’s disease as portrayed in a graphic novel^20^, two novels about Parkinson’s disease^21,22^, a non-fiction book^23^ and a first-person illness narrative^24^. Erving Goffman’s theory of stigma was used to study whether the metaphors of Parkinson’s disease indicate loss of autonomy and stigmatization^25^. Third, metaphors in the patient narrative, novels, and non-fiction work were reviewed and a list of themes or categorizations common to 2 of the metaphors was generated (Table 1). Collectively, this study aims to illustrate that the metaphors that are used to imagine the attributes of Parkinson’s disease (such as a stooped posture, shuffling gait, facial masking, and trembling) can influence treatment compliance and affect quality of life^26^.

**Table 1:** Metaphors and figurative language theme descriptions used in the contemporary story and -drawing from a person with Parkinson’s disease and within the two novels, graphic novel and non-fiction work.

Theme	Description	*N* (%) of phrases categorized into theme	Examples of metaphors/figurative language
Extreme experience	Specific scenarios detailing an emotionally salient circumstance/ action	19 (24.4%)	Setback, contagious disease, atomic bomb, fall by mischance, adventure in the woods, eternities in the space, trapped in that space, tumult, tantrum of selfish misery, on the verge of derailing, caged, deprived, a bottomless darkness and unreality, a nothingness, castrated by my illness, have run out of space to move in, learned habits are a liability, careering toward a crash, a let-up from the torture,
(Fictional) character	Characterization of a symptom or phenomena	12 (15.4%)	Clown, old iron horse, are you your brain, you the thought itself, furrowed organ guarded inside the cranium, trove, Rilke’s panther, a brain in conflict with itself, bad children, unreasoning two-year-olds, offending limb, bastards.
Dissociation or disconnect	Disconnect within the body or between the body and external surrounding	4 (5.1%)	Unexplained phenomenon, Parkinson’s prism, no guarantee, driving on the wrong side of the road.
Material/military/mechanical	Military or mechanical objects	4 (5.1%)	Ball of wool, colored strings, weapon, getting on a train
Miscellaneous objects/animal	Miscellaneous objects used to describe experience	2 (2.6%)	Wheat seedling, bad signals disrupt communication

Themes were partly derived from the study by Chahine LM et al. Neurol Clin Pract. 2021 Aug;11(4):e462-e471.

## Results

### Metaphors of self-perceived autonomy and stigma in the patient interview

At the beginning of the interview, the patient stated that Parkinson’s disease was a diagnosis he had expected, but never dared to directly address:

*“The symptoms have been there for 5 years and still make me feel like a clown. To me these years were feeling like a total loss of control. You can compare this to like getting on a train, a train that won’t ever stop”*.

In the beginning he thought of ways to hide the physical expression of disease symptoms:

*‘I try to disguise the symptoms (of which you say it’s Parkinson’s disease) by degrading them as a nasal cold in which the way you breathe has a huge impact. By breathing I have also learned how to kill the trembling. For me this is a way to silence it and the only way I can control it.’*

When we asked for the impact of these symptoms, the patient argued that:

*‘My left arm however feels sort of damaged, because of this trembling. It doesn’t matter anymore what I do with it. When I want my hand to completely stop shaking though, I’ll sit on it, or hit it against a wall till it starts to hurt.’*

The reasons why the patient tried to hide these symptoms were as follows:

*“Parkinson’s disease is famous for causing many setbacks (I constantly must start over again, for instance when my writing goes wrong) and thereby it is playing its negative role. Moreover, in my experience people consider Parkinson’s disease a contagious disease*.

When asked for reasons why W.B. thought people consider Parkinson’s disease a contagious disease, he mentioned the following:

*“I can notice this upon looking at the people around me, who are acting in a similar negative way when seeing me. When I go for shopping groceries, people keep looking at the shaking of my arm or leg. In fact, people seem to talk about Parkinson’s disease like it’s the 'atomic bomb', as I heard my neighbors doing. However, I think Parkinson’s disease is an unexplained phenomenon that you cannot describe with a few words.”*

Following multiple and increasingly repetitive episodes of the clinical encounter during which dopamine treatment and the side effects of treatment were discussed, the patient finally decided he did not want to be treated:

*“The medical jargon and my daily experience are at odds with each other. With this jargon, medical doctors happen to have a weapon. But for patients however, these words create a huge distance between them and this experience. This is a similar setback as we talked about earlier.”*

The patient mentioned that being in a hospital felt as being ‘alien to both mind and body’. In dealing with the symptoms, he stated that:

*“The moment you think you have found a way to deal with it, you’ll immediately feel standing at the start again. Whatever words you choose to describe it, it never feels complete*.

**Fig. 1:**
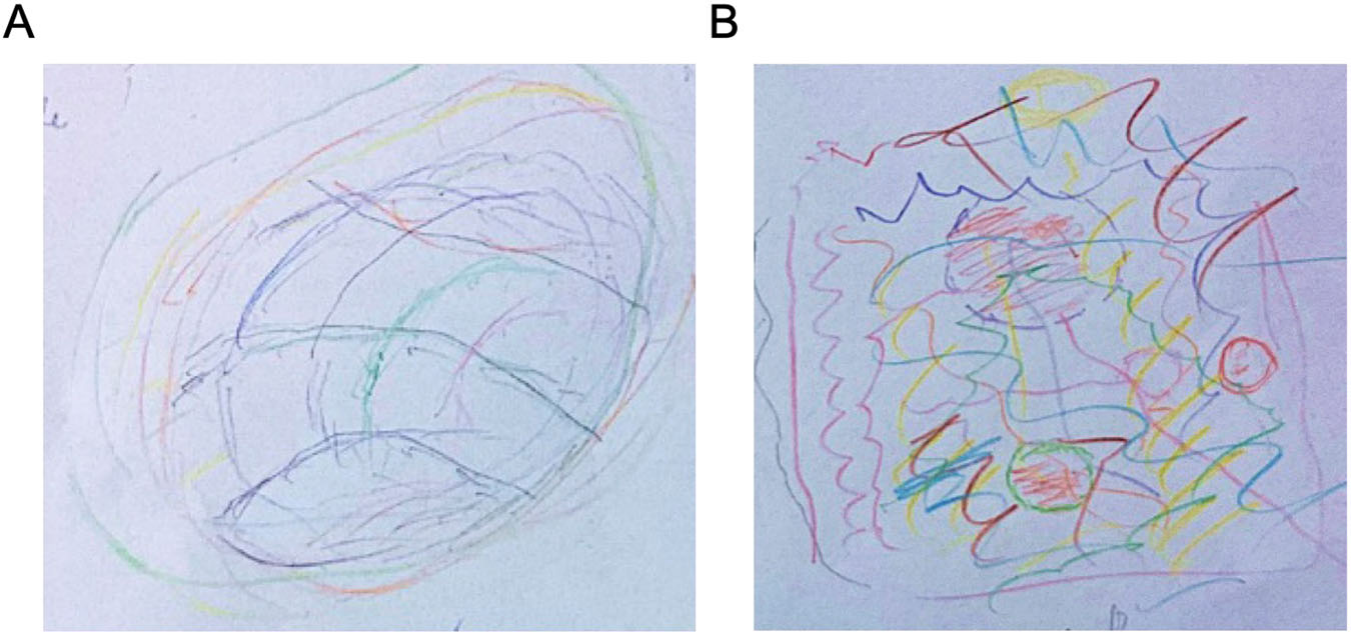
Drawing depicting the Parkinson’s disease experience. **A** Visual representation of the Parkinson’s disease experience as provided by the interviewed person with Parkinson’s disease (W.B.) who imagined himself to be a ball of wool made up of many different colored strings. These strings each represent a connection in his being as a person related to self-perceived autonomy and the world around him. **B** Following Parkinson’s disease these connections were being torn apart, represented in his illustration as the strings being spread chaotically across the floor.

In order to express himself more completely next to the metaphors as depicted in Table 1, the patient chose to draw his visual representation of the illness experience. In his drawing, he imagined himself to be a ball of wool made up of many different colored strings (Fig. 1A). These strings each represent a connection in his being as a person, related to self-perceived autonomy and the world around him. These connections were being torn apart, represented in his drawing as the strings being spread chaotically across the floor (Fig. 1B). In addition (and in line with work from narrative medicine scholar Rita Charon, who advocated that ‘patients should be the curators of what we write about them’^4^) we asked the patient to comment on Jonathan Franzen’s *The Corrections* and Peter Dunlap-Shohl’s *My Degeneration: A Journey Through Parkinson’s.* He stated in these comments the following:

*‘What I do miss in Jonathan Franzen’s story is that the correction can be greater than Parkinson’s disease. Rage about my hand and arm also arise in me, but I can only remain a spectator trying to do the opposite of what Parkinson’s does. I find the drawing in* Fig. 3*harsh and explicit because this face looks too frightened. There is no beauty in Parkinson’s disease, but there can be beauty in me as a person. I am a clown with Parkinson’s disease, but one with a smile, knowing my hand is moving in the wrong direction.'*

### Drawings and metaphors pinpointing loss of autonomy and self-perceived stigma in the graphic novel “My Degeneration” and the non-fiction book ‘Awakenings’ by Oliver Sacks

**Fig. 2:**
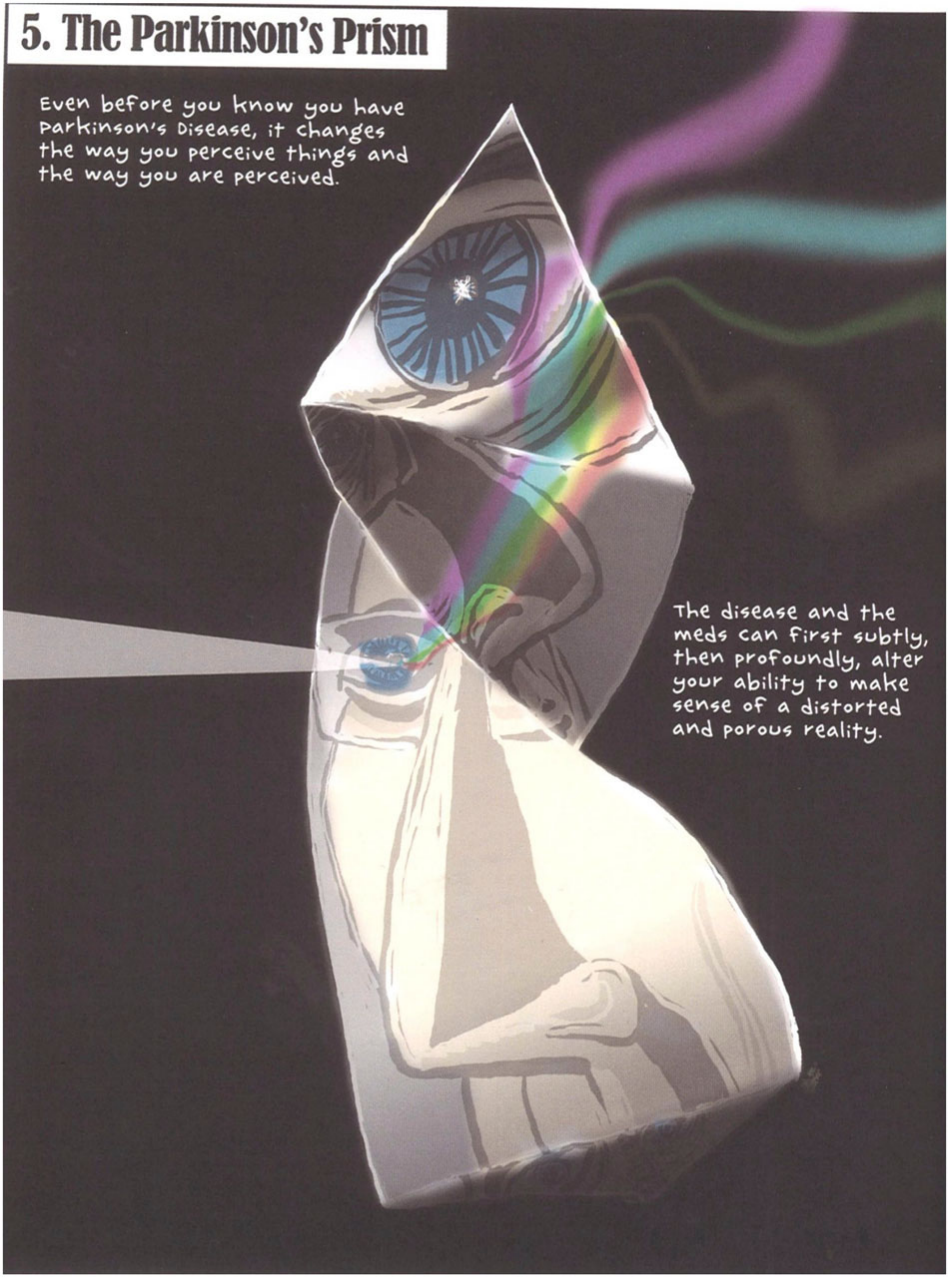
The ‘Parkinson prism’. The ‘Parkinson prism’ is a metaphor that demonstrates how disease and medication alter a person’s ability to make sense of a distorted and parous reality. Image derived from the graphic novel My Degeneration: A Journey Through Parkinson’s by Peter Dunlap-Shohl.

**Fig. 3:**
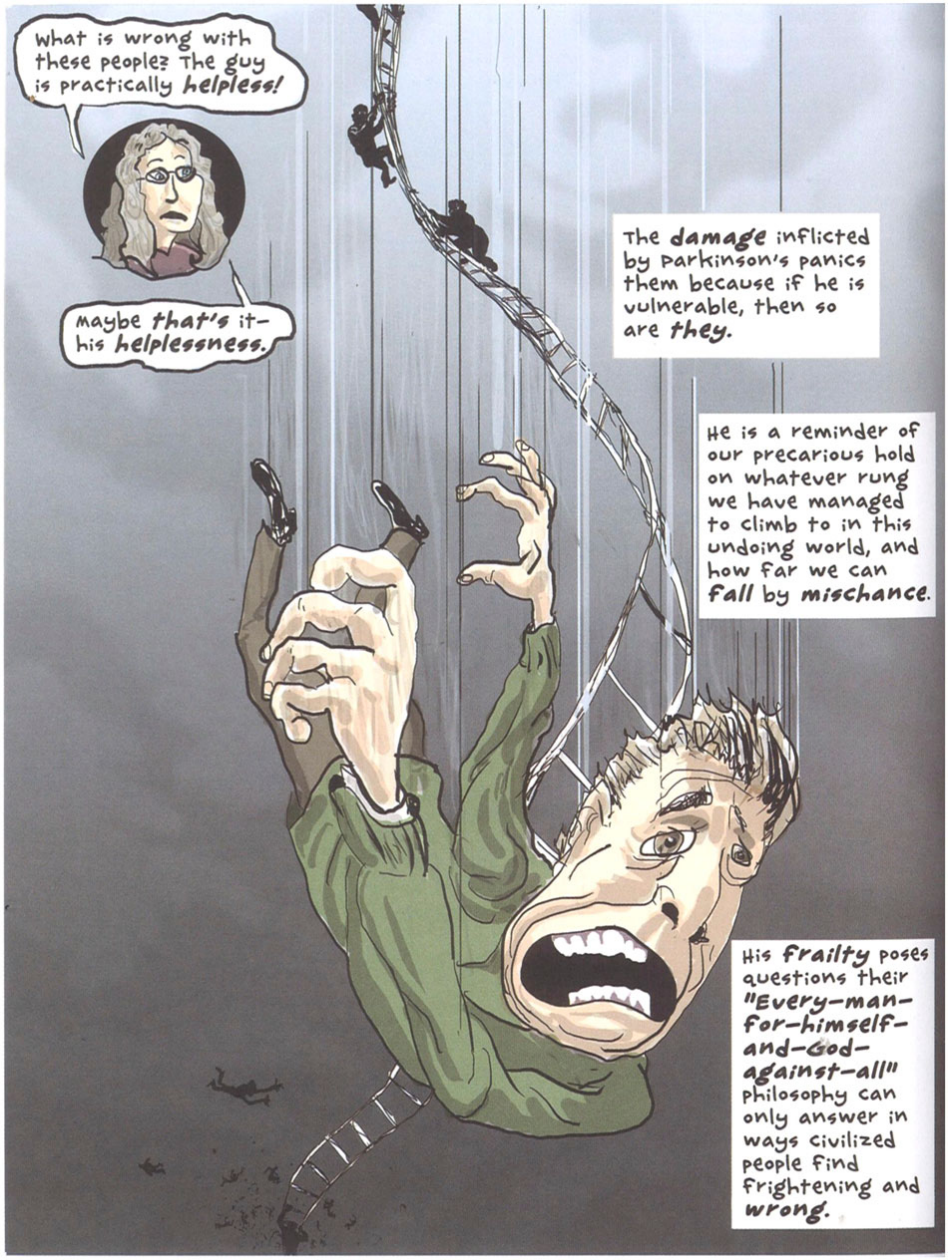
Fall by mischange. The damage inflicted by Parkinson’s disease is a reminder of how far we can fall by mischance. Image derived from the graphic novel My Degeneration: A Journey Through Parkinson’s by Peter Dunlap-Shohl.

To study the Parkinson’s disease experience in cartoon drawings, we selected two cartoon drawings from the graphic novel “My Degeneration”. Figure 2 depicts the ‘Parkinson’s prism’, a metaphor that demonstrates how disease alters a person’s ability to make ‘sense of a distorted and parous reality’ (p. 50). Therefore, the damage inflicted by Parkinson’s disease is a reminder of ‘how far we can fall by mischance’. This is depicted in another cartoon shown in Fig. 3. This cartoon states that the frailty that comes with this disease poses questions ‘philosophy can only answer in ways civilized people find frightening’ (p. 58).

To further analyze the meaning of the ‘Parkinson’s prism’, we assessed “Awakenings” by Oliver Sacks (1973). This is an account of twenty patients who survived the 1920s encephalitis lethargica epidemic and developed post-encephalitic parkinsonism with symptoms ‘more varied and severe than those seen in common Parkinson’s disease’ (p. 14). When Sacks asked one of his patients, Leonard L., ‘What’s it like being the way you are? What would you compare it to?’, Leonard L. replied: ‘Caged. Deprived. Like Rilke’s “Panther”^23^ (p. 205)’ “There’s an awful presence,” he once tapped out, ‘and an awful absence. The presence is a mixture of nagging and pushing and pressure, with being held back and constrained and stopped – I often call it ‘the goad and halter’. The absence is a terrible isolation and coldness and shrinking – more than you can imagine, Dr. Sacks, much more than anybody who isn’t this way can possibly imagine – a bottomless darkness and unreality.” (…) “At other times,’ Mr. L. would tap out, ‘there’s none of this sense of pushing or active taking away, but a sort of total calmness, a nothingness, which is by no means unpleasant. It’s a let-up from the torture. On the other hand, it’s something like death. At these times I feel I’ve been castrated by my illness, and relieved from all the longings other people have”^23^ (p. 205).

More insight into the world of Parkinson’s disease is offered by Francis D, who is one of those patients that describe a fantastical-mathematical world remarkably similar to that which faced ‘Alice’. Miss D. pinpoints the fundamental distortions of Parkinsonian *space*, with her difficulties with ‘angles, circles, sets, and limits'. She once said of her ‘freezing’: *‘It’s not as simple as it looks. I don’t just come to a halt, I am still going, but I have run out of space to move in … You see, my space, our space, is nothing like your space: our space gets bigger and smaller, it bounces back on itself, and it loops itself round till it runs into itself*^23^ (p. 339).

### Metaphors of self-perceived autonomy and stigma in the novel ‘The Corrections’ by Jonathan Franzen

To further study the world of Parkinson’s disease we assessed the novel “*The Corrections”* by Jonathan Franzen (2001). Herein we follow a protagonist with Parkinson’s disease named Alfred Lambert, a retired railroad engineer depicted with his family as His symptoms of disease particularly include a change in his perception of time:

*(…) “the problem of existence was this: that, in the manner of a wheat seedling thrusting itself up out of the earth, the world moved forward in time by adding cell after cell to its leading edge, piling moment on moment, and that to grasp the world even in its freshest, youngest moment provided no guarantee that you’d be able to grasp it again a moment later.” (p. 66)*

These time-altering events experienced by Albert, manifest themselves in the way language is processed. This way Albert’s ‘Parkinson’s prism’ illustrates how language and being cannot be understood apart from one another:

*“He began a sentence: “I am - but when he was taken by surprise, every sentence became an adventure in the woods (…) but in the instant of realizing he was lost, time became marvelously slow and he discovered hitherto eternities in the space between one word and the next, or rather he became trapped in that space between words and could only stand and watch as time sped on without him (…)” (p. 11)*

In fact, the ongoing characteristics associated with Parkinson’s disease, seem to amplify ‘Parkinson’s prism’ effect on the self as narrator. Particularly because the trembling of both hands make Alfred feel betrayed:

*“These shaking hands belonged to nobody but him, and yet they refused to obey him. They were like bad children. Unreasoning two-year-olds in a tantrum of selfish misery. (..) (p. 67)*.

Similarly, as our patient was arguing, Alfred also thought of a way to hide the physical expression of disease symptoms because ‘Parkinson’s prism’ altered Alfred’s ability to make ‘sense of a distorted and parous reality’:

*“Alfred took pleasure in thinking about chopping his hand off with a hatchet. He wanted to let the offending limb know just how angry he was. If only to show how little he cared for it when it insisted on disobeying him. It brought a kind of ecstasy to imagine the first deep bite of the hatchet’s blade into bone and muscle. But alongside his ecstasy there was also the unshakable feeling of despair. He and his hand had been together for his entire life after all. (p. 67)”*

*“He would have liked to remove his legs entirely. They were weak and restless and wet and trapped. He kicked a little and rocked in his unrocking chair. His hands were in a tumult. The less he could do about his legs, the more he swung his arms. The bastards had him now, he’d been betrayed, and he began to cry. If only he’d known! If only he’d known, he could have taken steps, he’d had the gun, he’d had the bottomless cold ocean, if only he’d known.” (p. 555)*

Erving Goffman has argued that stigma particularly results from the eyes of others: “While the stranger is present before us, evidence can arise of his possessing an attribute that makes him different from others… and of a less desirable kind – in the extreme, a person who is quite thoroughly bad, or dangerous, or weak” ^25(p. 12)^. Likewise, Alfred is seen in the eyes of his son:

*“Alfred seemed forever on the verge of derailing as he lurched down hallways or half slid downstairs or wolfed at a sandwich from which lettuce and meat loaf rained; checking his watch incessantly (..) the old iron horse was careering toward a crash, and Gary could hardly stand to look.” (p. 171)*

For social situations, Goffman has argued that ‘with an individual known or perceived to have a stigma, we are likely, then, to employ categorizations that do not fit, and we and he are likely to experience uneasiness’ ^25(p. 31)^. Also, in dealing with bladder dysfunction, another illustration is given of the way Alfred is being frowned upon by others, and how this impacts self-perceived autonomy and stigma:

*“The problem was that his nervous system could no longer be relied on for an accurate assessment of his need to go. At night his solution was to wear protection. By day his solution was to visit a bathroom hourly and always to carry his old black raincoat in case he had an accident to hide.” (p. 329)*

### Metaphors of self-perceived autonomy and stigma in the novel ‘Elena Knows’ by Claudia Pińeiro and in John Palfreman’s The Bright Side of Parkinson’s

To further understand how the symptoms of Parkinson’s disease can create stigma, we studied how the main protagonist (Elena) in *Elena Knows* deals with disease symptoms. Elena is a 63-year-old woman with Parkinson’s disease who is trying to deal with the basic aspects of living a normal life, like “Just walking, to get to the ten o’clock train.” (p. 7)

*“From her position, seated, she tries to lift her foot in the air, the foot now responds to the message and rises. So she’s ready, she knows. She places her palms on her seated thighs, she puts her two feet together so that her knees are at ninety-degree-angles, then she crosses her right hand to her left shoulder and her left hand on her right shoulder, she begins to rock back and forth on the chair and then, with the momentum, she stands up.” (p. 7)*

Characteristics associated with the disease, like the shuffling gait or the stooped posture, instigate a self-perceived sense of stigmatization and disapproval from others. Goffman has argued that this: “(..) is called a stigma, especially when its discrediting effect is very extensive. Sometimes it is also called a failing, a shortcoming, a handicap. It constitutes a special discrepancy between virtual and actual social identity”^25(p. 12 - 13)^. Likewise, Elena questions her self-perceived sense of identity:

*‘What’s left of you when your arm can’t even put on a jacket and your leg can’t even take a step and your neck can’t straighten up enough to let you show your face to the world, what’s left? Are you your brain, which keeps sending out orders that won’t be followed? Or are you the thought itself, something that can’t be seen or touched beyond that furrowed organ guarded inside the cranium like a trove?’ (p. 72)*

When disease symptoms progress into a failure to swallow with normal frequency, Elena starts to experience the reaction of others. As such the novel puts particular emphasis on the experience of social interaction. This could, like Goffman has argued, make us, “believe the person with a stigma is not quite human. On this assumption we exercise varieties of discrimination, through which we effectively, if often unthinkingly, reduce his life chances”^25(p. 15)^. This way, Elena (believed to have less chance in life) is contrasted with a woman and her young daughter as follows:

*‘A woman and her daughter sit on the bench next to Elena. The girl’s feet don’t reach the ground, Elena watches her swing them in the air. She knows the girl is looking at her. She knows that she leans over to her mother and whispers something in her ear. I’ll tell you later, the mother says, and the girl swings her legs faster than before.’*

Goffman states that ‘the stigmatized individual is likely to feel that he is ‘on’, having to be self-conscious and calculating about the impression he is making, to a degree and in areas of conduct which he assumes others are not.’ (p. 25). In his New York Times first-person illness narrative, John Palfreman argues that this state of being self-conscious and calculating about the impression an individual with Parkinson’s disease must make is because:

*‘People with Parkinson’s progressively lose core pieces of themselves. We forget how to walk. Our arm muscles get weaker. Our movements slow down. Our hands fumble simple tasks like buttoning a shirt or balancing spaghetti on a fork. Our faces no longer express emotions. Our voices lose volume and clarity. Our minds, in time, may lose their sharpness and more’*^24^.

In the beginning of his essay, Palfreman argues that what is responsible for this, is something that ‘*keeps sending out orders that won’t be followed’*:

*These bad signals disrupt communication between the brain and the muscles. This is one reason people with Parkinson’s have trouble picking up small objects and moving around fluently: Their motions are too hesitant, too small, too slow, too rigid, too shaky, too feeble, and badly timed. These are symptoms of a brain in conflict with itself*^24^.

In Palfreman’s view, Parkinson’s disease disrupts the narrative coherence of the stories told by persons with Parkinson’s disease because, *‘When the production of dopamine is interrupted, as it is with Parkinson’s, the signals passing through the basal ganglia are garbled, and it ends up giving poor advice.’*’This is because:

*‘Having Parkinson’s feels a bit like going on vacation in another country and having to drive on the “wrong” side of the road. Driving is one of those activities that we outsource, in large part, to the basal ganglia. When an American, who has spent thousands of hours driving on the right side of the street, tries to drive in England, his learned habits are a liability’*^24^.

## Discussion

This narrative medicine perspective illustrates that the attributes of Parkinson’s disease activate can activate stigma. This is an experience expressed in metaphors imagining extreme experiences (24.4%) like a ‘fall by mischance’, a ‘tantrum of selfish misery’ or a ‘bottomless darkness and unreality’ (Table 1). Collectively, these metaphors pinpoint loss of autonomy thereby influencing many factors, including social interactions, treatment adherence, and even the decision to initiate treatment or not. As the selected cartoons from *My Degeneration* demonstrate, this narrative instigates a way of seeing which is metaphorically speaking called a ‘Parkinson’s prism’. How this 'prism' is perceived in the patient narrative is illustrated in metaphors that make Parkinson’s disease ‘a contagious disease’, experienced as a setback and impacting like an ‘atomic bomb’. Similarly, in Oliver Sacks’ account of Leonard L, parkinsonism is like being ‘Caged’ and ‘Deprived’, an experience that feels for John Palfreman as *‘having to drive on the “wrong” side of the road’*.

In this study the aforementioned metaphors of Parkinson’s disease pinpoint loss of autonomy and stigma, because the metaphors depict extreme experiences of losing control (Table 1). More examples are the drawing by the interviewed patient illustrating ‘strings being spread chaotically’ (Fig. 1A) and the cartoon depicting a ‘fall by mischance’ (Fig. 3). These selected metaphors, and particularly the patient narrative, are *chaos stories* because “(…) the body telling chaos stories defines itself as being swept along, without control, by life’s fundamental contingency”^5^. (p. 102). Arthur Frank has argued that ‘In the chaos narrative, consciousness has given up the struggle for sovereignty over its experience’^5^ (p. 104) This way Parkinson’s disease, and the prism it instigates, disrupt a person’s narrative coherence. As a consequence, ‘People living these stories regularly accuse medicine of seeking to maintain its pretense of control (…) at the expense of denying the suffering of what it cannot treat’^5^. (p. 98). This could impact factors such as physician-patient interaction, as was demonstrated by a study into the metaphors used by 86 individuals with Parkinson’s disease for whom their figurative language was not uniformly interpreted and understood by neurologists^27^.

Interestingly, for the interviewed patient, medical jargon is perceived as a very alternative set of ‘words that can be used as a weapon’ to create a ‘huge distance’ between medical doctors and the Parkinson’s disease experience. This could suggest denial of the chaos story as told by the patient because, as Frank has pointed out, ‘Denials of the chaos narrative often begin with the listener asserting how, in such circumstances, he would find some way out’ (…) All of us on the outside of some chaos want assurances that if we fell in, we could get out. But the chaos narrative is beyond such bargaining; there is no way out.’ (p. 102)^5^ Instead, precise and respectful terms to explain the disease course of Parkinson’s disease could contribute to symmetrical and not patronizing conversations between physicians and persons with Parkinson’s disease^28^. For the patient described here, an experience of the opposite resulted in his decision to refuse treatment, emphasizing that physician communication is an essential component of a patient’s adherence to medical advice^29^. We have recently argued that during the initial communications with someone diagnosed recently with Parkinson’s disease, the emphasis should not so much be on whether or not to start dopaminergic replacement therapy, but rather on discussing the concept of Hopamine: a personalized dose of hope and perspective that is unique to each individual^30^. Whereas most physicians and other medical physicians place disproportionate emphasis on initiating a battery of medical interventions, affected individuals might be helped in a better way by discussing what matters to them most.

Interestingly, in a detailed linguistical analysis of the novel *The Corrections*, the perception of stigma following a diagnosis of Parkinson’s disease was confirmed in words like. Particularly a sense of ‘betrayal and disconnection’ that described the experience by the main protagonist Alfred^31^. Since Gofman’s work, it has become clear that in individuals with Parkinson’s disease, stigma particularly follows from severe motor symptoms^32,33^. It develops mainly in unfamiliar places, at the working place or in contact with people without Parkinson’s disease^34^. In certain areas of the world, stigma takes a completely different form, for example in a country such as South Africa, where Parkinson’s disease is regarded as a result from witchcraft, forcing affected individuals to be banned from society^35^. Because of stigma and in response to the symptoms of Parkinson’s disease, Alfred in *The Corrections* is found wanting to inflict harm upon himself. This was similarly suggested in the story of the patient presented here who mentioned that ‘*When I want my hand to completely stop shaking though, I’ll (…) hit it against a wall till it starts to hurt.’* Likewise, in *My Degeneration*, the feeling of betrayal is depicted as a man in a suit, hell-bent on making the patient’s life as miserable as possible. The main protagonist in *My Degeneration* therefore muses on Parkinson’s disease being “cunning and mysterious” and him subtly taking away all that he has learned during his lifetime. This way, Parkinson’s disease is presented as a man subtly diminishing the main protagonists’ ability to self-govern^36^. This experience of losing the ability to self-govern, follows from the experience of sensory and perceptual deficits which facilitate physical misperceptions^37^. Interestingly, narrative medicine intervention studies, that aimed at the rehabilitation of these misperceptions using drawing tasks, have been found to improve these mental representations^38^. Also, *My Degeneration* was used to facilitate this improvement, as was demonstrated by clinicians working in a multidisciplinary movement disorders clinic in the USA^39^. Close reading of *My Degeneration* followed by the assessment of pre- and post-reading attitudes and degree of empathy identified that significant improvement was observed after reading and discussing the graphic novel in group sessions.

In the context of narrative medicine, a wide range of research methodologies is used compared to the ones used in biomedicine. In addition to quantitative research methods, qualitative approaches produce rich knowledge: drawings by patients of their illness and the impact of an illness on daily life (for a systematic review, see Ref. ^19^). Drawing of an illness not only serves diagnostic purposes into the subjective response to an illness, but intervention studies have shown how changes in patient drawings can identify medical outcomes^40^. In persons with Parkinson’s disease, drawings have been applied in order to examine its lived experience^41^. Moreover, adjusting to living with Parkinson’s disease was reviewed in a meta-ethnography of qualitative research, emphasizing the contribution of qualitative methods for biopsychosocial research in people with Parkinson’s disease^42^. Particularly ‘first-person illness narratives’ provide great opportunity for research into the practice of ‘thinking with stories. Arthur Frank has pointed out that for first-person illness narratives this is not the ‘illusion of authorial presence’ within the text^43^. Instead, the reader should ‘recognize the real presence in the textual absence’ because illness is an ‘absence from the social presence that health takes for granted, and this absence is the reflexive ground of the illness narrative’^43^ In contrast, the textual effect of literary fiction, according to Frank, should not be ‘a form of distancing between the writers of such narratives and those readers who need, for different reasons, to affirm what the narrator needs to show them’^43^. In our view, the selected works of Jonathan Franzen, Claudia Piñeiro, and Oliver Sacks are very well equipped to avoid a distancing from the Parkinson’s disease experience. Nonetheless, future studies should investigate how textual differences in first-person illness narratives compared to works of art (such as literary fiction) improve the practice of ‘thinking with stories’.

In addition to the aforementioned examples, other illustrations of narrative medicine in clinical practice are storytelling^44^ and creative writing sessions in medical education^7^. This way, narrative medicine was found to improve competencies such as relationship-building, empathy, and confidence^45^. Regarding its use to promote treatment success, narrative medicine sessions in neuro-oncology practice with persons with brain cancer (who shared their stories of loss of self-identity during illness and treatment) could provide visible contributions of disease impact, thereby helping the treatment team to assess patient needs^46^. Whether it could reduce self-perceived autonomy and stigma in Parkinson’s disease, thereby improving decision making in its treatment (like the person presented in this report) remains to be studied. These studies should select persons with divergent characteristics, because particular examples such as being a male patient significantly contributes to self-stigma^3^. Moreover self-stigma^47^ and mental health significantly impact self-esteem^48^ and the experienced quality of life^26^. This is exacerbated by the common tendency of persons with Parkinson’s disease to start avoiding social situations, such as the professional working place, dining in public places or even the clinical encounter.

In conclusion, this narrative medicine perspective illustrates that the attributes of Parkinson’s disease make the patient feel different from others, thereby affecting self-perceived autonomy and stigma. These factors can have a great impact on social interactions and can even influence crucial treatment decisions, thereby also affecting quality of life. We also emphasize how narrative medicine can help to focus the medical consultations with affected individuals and their families on issues that matter most to them, rather than on the medical perspective, well-intended as this may be. As such, narrative medicine is a hitherto underutilized yet critical component of the much-needed move towards personalized medicine, an approach achieved through the reciprocity of thinking with stories.

## Methods

### Patient interview and drawing of the Parkinson’s disease experience

The patient (W.B.) was interviewed following inpatient admission to our hospital in September 2022. W.B. had been diagnosed with Parkinson’s disease 3 years earlier and required inpatient admission although he refused any treatment. The patient provided written informed consent, both for the interview to be conducted and published. (The Medical Research Involving Human Subjects Act (WMO) in The Netherlands is applicable when persons in scientific research are subjected to treatment or are required to behave in a particular way. If a study falls under the scope of the WMO because these requirements are applicable, it must undergo a review by an accredited Medical Review Ethics Committee (MREC). However, the interviewed patient was a mentally competent citizen who was neither subjected to treatment or an intervention nor was he asked to follow a behavioral strategy as referred to in the WMO. Therefore, the Dutch law (WMO) regulating medical research in humans stipulates that this study is exempt from any requirement regarding approval by regulating bodies.)

### Identification of (graphic)-novels and non-fiction related to Parkinson’s disease

We aimed to identify a selection of (graphic)-novels and non-fiction (books) where metaphors of *Parkinson’s disease* represent a meaningful and substantial component. After a search in the LITMED Literature Arts Medicine Database of the Division of Medical Humanities at NYU Langone Health [https://medhum.med.nyu.edu/], we selected the graphic novel *“My Degeneration: A Journey Through Parkinson’s”* by Peter Dunlap-Shohl^20^, the novels *“The Corrections”* by Jonathan Franzen^22^ and *“Elena Knows”* by Claudia Piñeiro^21^. In addition, we also included the non-fiction book *“Awakenings”* by Oliver Sacks^23^ and the first-person illness narrative *“The Bright Side of Parkinson’s”* by Jon Palfreman^24^. This method of studying (graphic)-novels and non-fiction (books) as a source to review how a particular illness is represented in literary works is a validated and accepted methodology^8^, and previously used in a comparison with empirical studies on patients’ representations of the particular illness^11^ or to debate a diagnosis or therapeutic approach^49^.

### Qualitative analysis and interpretation of metaphors

Because metaphors cannot always be translated across languages or cultures (and to overcome a potential ‘loss in translation’) we asked a native speaker in both Dutch and American English to translate the patient story. The use of metaphors was reviewed and a list of themes common to at least two of the metaphors was generated. This method was previously used in a study among individuals with Parkinson’s disease imagining their “off” periods^27^. Classifications were reviewed by authors B.W.F., R.K., A.A.K., and B.R.B., and final classifications were chosen after discussion.

### Theoretical model to analyze the Parkinson’s disease experience

Using Erving Goffman’s theory of the stigma relationship as outlined in the book *Stigma: Notes on the Management of Spoiled Identity*, we selected quotes from the identified (graphic)-novels and non-fiction works that were related to the loss of autonomy and the experience of stigma. These quotes were compared to excerpts from the patient story and the drawing of the illness experience.

### Reporting summary

Further information on research design is available in the Nature Research Reporting Summary linked to this article.

### Supplementary information

Reporting Summary

## Data Availability

The datasets used and/or analyzed during the current study are available from the corresponding author on reasonable request.

## Code availability

Code sharing is not applicable to this article as no custom computer code or scripts were generated or used during the current study.
