# Supplementary material for: Narrative medicine pinpoints loss of autonomy and stigma in Parkinson’s disease
Source: NPJ Parkinsons Dis. 2023 Nov 1;9:152. doi: 10.1038/s41531-023-00593-y (PMC10620172; doi:10.1038/s41531-023-00593-y)
Supplement: Supplementary file 1 — Reporting Summary [file 41531_2023_593_MOESM1_ESM.pdf]

nature portfolio

Corresponding author(s): B.W. Florijn

Last updated by author(s): 13th april 2023

Reporting Summary

Nature Portfolio wishes to improve the reproducibility of the work that we publish. This form provides structure for consistency and transparency in reporting. For further information on Nature Portfolio policies, see our Editorial Policies and the Editorial Policy Checklist.

Statistics

For all statistical analyses, confirm that the following items are present in the figure legend, table legend, main text, or Methods section.

n/a	Confirmed	
☒	☐	The exact sample size (*n*) for each experimental group/condition, given as a discrete number and unit of measurement
☒	☐	A statement on whether measurements were taken from distinct samples or whether the same sample was measured repeatedly
☒	☐	The statistical test(s) used AND whether they are one- or two-sided<br>*Only common tests should be described solely by name; describe more complex techniques in the Methods section.*
☒	☐	A description of all covariates tested
☒	☐	A description of any assumptions or corrections, such as tests of normality and adjustment for multiple comparisons
☒	☐	A full description of the statistical parameters including central tendency (e.g. means) or other basic estimates (e.g. regression coefficient) AND variation (e.g. standard deviation) or associated estimates of uncertainty (e.g. confidence intervals)
☒	☐	For null hypothesis testing, the test statistic (e.g. *F*, *t*, *r*) with confidence intervals, effect sizes, degrees of freedom and *P* value noted<br>*Give P values as exact values whenever suitable.*
☒	☐	For Bayesian analysis, information on the choice of priors and Markov chain Monte Carlo settings
☒	☐	For hierarchical and complex designs, identification of the appropriate level for tests and full reporting of outcomes
☒	☐	Estimates of effect sizes (e.g. Cohen's *d*, Pearson's *r*), indicating how they were calculated

Our web collection on statistics for biologists contains articles on many of the points above.

Software and code

Policy information about availability of computer code

Data collection	NA
Data analysis	NA

For manuscripts utilizing custom algorithms or software that are central to the research but not yet described in published literature, software must be made available to editors and reviewers. We strongly encourage code deposition in a community repository (e.g. GitHub). See the Nature Portfolio guidelines for submitting code & software for further information.

Data

Policy information about availability of data

All manuscripts must include a data availability statement. This statement should provide the following information, where applicable:

- Accession codes, unique identifiers, or web links for publicly available datasets
- A description of any restrictions on data availability
- For clinical datasets or third party data, please ensure that the statement adheres to our policy

The datasets used and/or analysed during the current study available from the corresponding author on reasonable request.

Research involving human participants, their data, or biological material

Policy information about studies with human participants or human data. See also policy information about sex, gender (identity/presentation), and sexual orientation and race, ethnicity and racism.

Reporting on sex and gender	NA
Reporting on race, ethnicity, or other socially relevant groupings	NA
Population characteristics	NA
Recruitment	The patient (W.B.) was interviewed following inpatient admission to our hospital in September 2022. The patient provided written informed consent, both for the interview to be conducted and published.
Ethics oversight	The Medical Research Involving Human Subjects Act (WMO) in The Netherlands is applicable when persons in scientific research are subjected to treatment or are required to behave in a particular way. If a study falls under the scope of the WMO because these requirements are applicable, it must undergo a review by an accredited Medical Review Ethics Committee (MREC). However, the interviewed patient was a mentally competent citizens who was neither subjected to treatment or an intervention nor was he asked to follow a behavioral strategy as referred to in the WMO. Therefore, the Dutch law (WMO) regulating medical research in humans stipulates that this study is exempt from any requirement regarding approval by regulating bodies.

Note that full information on the approval of the study protocol must also be provided in the manuscript.

Field-specific reporting

Please select the one below that is the best fit for your research. If you are not sure, read the appropriate sections before making your selection.

☐ Life sciences ☒ Behavioural & social sciences ☐ Ecological, evolutionary & environmental sciences

For a reference copy of the document with all sections, see nature.com/documents/nr-reporting-summary-flat.pdf

Behavioural & social sciences study design

All studies must disclose on these points even when the disclosure is negative.

Study description	Case report. Qualitative and quantitative data.
Research sample	Single patient.
Sampling strategy	NA
Data collection	The use of metaphors was reviewed and a list of themes common to at least two of the metaphors was generated. Classifications were reviewed by authors B.W.F., R.K., A.A.K., and B.R.B., and final classifications were chosen after discussion.
Timing	Research conducted during one month (september 2022)
Data exclusions	NA
Non-participation	NA
Randomization	NA

Reporting for specific materials, systems and methods

We require information from authors about some types of materials, experimental systems and methods used in many studies. Here, indicate whether each material, system or method listed is relevant to your study. If you are not sure if a list item applies to your research, read the appropriate section before selecting a response.

Materials & experimental systems

n/a	Involved in the study
☒	☐ Antibodies
☒	☐ Eukaryotic cell lines
☒	☐ Palaeontology and archaeology
☒	☐ Animals and other organisms
☒	☐ Clinical data
☒	☐ Dual use research of concern
☒	☐ Plants

Methods

n/a	Involved in the study
☒	☐ ChIP-seq
☒	☐ Flow cytometry
☒	☐ MRI-based neuroimaging

Plants

Seed stocks

NA

Novel plant genotypes

NA

Authentication

NA
